# Time‐domain heart rate variability features for automatic congestive heart failure prediction

**DOI:** 10.1002/ehf2.14593

**Published:** 2023-11-27

**Authors:** Jeban Chandir Moses, Sasan Adibi, Maia Angelova, Sheikh Mohammed Shariful Islam

**Affiliations:** ^1^ School of Information Technology Deakin University Burwood VIC 3125 Australia; ^2^ Aston Digital Futures Institute, College of Physical Sciences and Engineering Aston University Birmingham UK; ^3^ Institute for Physical Activity and Nutrition (IPAN) Deakin University Burwood VIC 3125 Australia

**Keywords:** Heart failure, Heart rhythm, Time‐domain, Machine learning, Prediction

## Abstract

**Aims:**

Heart failure is a serious condition that often goes undiagnosed in primary care due to the lack of reliable diagnostic tools and the similarity of its symptoms with other diseases. Non‐invasive monitoring of heart rate variability (HRV), which reflects the activity of the autonomic nervous system, could offer a novel and accurate way to detect and manage heart failure patients. This study aimed to assess the feasibility of using machine learning techniques on HRV data as a non‐invasive biomarker to classify healthy adults and those with heart failure.

**Methods and results:**

We used digitized electrocardiogram recordings from 54 adults with normal sinus rhythm and 44 adults categorized into New York Heart Association classes 1, 2, and 3, suffering from congestive heart failure. All recordings were sourced from the PhysioNet database. Following data pre‐processing, we performed time‐domain HRV analysis on all individual recordings, including root mean square of the successive difference in adjacent RR interval (RRi) (RMSSD), the standard deviation of RRi (SDNN, the NN stands for natural or sinus intervals), the standard deviation of the successive differences between successive RRi (SDSD), the number or percentage of RRi longer than 50 ms (NN50 and pNN50), and the average value of RRi [mean RR interval (mRRi)]. In our experimental classification performance evaluation, on the computed HRV parameters, we optimized hyperparameters and performed five‐fold cross‐validation using four machine learning classification algorithms: support vector machine, k‐nearest neighbour (KNN), naïve Bayes, and decision tree (DT). We evaluated the prediction accuracy of these models using performance criteria, namely, precision, recall, specificity, F1 score, and overall accuracy. For added insight, we also presented receiver operating characteristic (ROC) plots and area under the ROC curve (AUC) values. The overall best performance accuracy of 77% was achieved when KNN and DT were trained on computed HRV parameters with a 5 min time window. KNN obtained an AUC of 0.77, while DT attained 0.78. Additionally, in the classification of severe congestive heart failure, KNN and DT had the best accuracy of 91%, with KNN achieving an AUC of 0.88 and DT obtaining 0.92.

**Conclusions:**

The results show that HRV can accurately predict severe congestive heart failure. The findings of this study could inform the use of machine learning approaches on non‐invasive HRV, to screen congestive heart failure individuals in primary care.

## Introduction

Congestive heart failure is a progressive clinical syndrome characterized by exercise intolerance and/or signs of congestion in the presence of a cardiac condition. It is associated with high morbidity and mortality rates.^1^, ^2^ The underlying causes of congestive heart failure include structural abnormalities of the heart, functional impairments, and various contributing factors, such as hypertension, valvular heart disease, uncontrolled arrhythmias, myocarditis, and congenital heart disease.^3^ Additionally, a significant issue associated with the severity of congestive heart failure is end‐stage heart failure.^4^

Globally, there has been a rapid increase in the prevalence of congestive heart failure and its associated health burden, particularly among older individuals and people living in low‐ to middle‐income countries.^5^ Currently, there are approximately 64.34 million cases of congestive heart failure worldwide, equivalent to 8.52 per 1000 inhabitants, contributing to a loss of 9.91 million years due to disability.^5^ Furthermore, congestive heart failure is known to present a range of complications, including arrhythmias (such as atrial fibrillation, ventricular arrhythmias, and bradyarrhythmia), thromboembolism (leading to conditions like stroke, peripheral embolism, deep venous thrombosis, and pulmonary embolism), gastrointestinal issues (including hepatic congestion, hepatic dysfunction, and malabsorption), musculoskeletal problems (such as muscle wasting), and respiratory challenges (involving pulmonary congestion, respiratory muscle weakness, and pulmonary hypertension).^6^

Heart failure, a prognostically severe syndrome, may remain undetected in over half of the cases, especially up to 76% of these undiagnosed cases involving patients with preserved ejection fraction.^7^ Traditionally, congestive heart failure is diagnosed by physicians upon the onset of symptoms through a combination of physical examination, a review of the patient's medical history, and various diagnostic tests. These tests include, but are not limited to, a complete blood count, urinalysis, a complete metabolic profile assessing serum electrolyte levels (including calcium and magnesium), blood urea nitrogen, serum creatinine, glucose levels, fasting lipid profile, liver function tests, and thyroid‐stimulating hormone evaluation to detect abnormal left ventricle and/or heart valve function.^4^, ^8^

The process of diagnosing congestive heart failure is often time‐consuming, requires specialized skills, and is associated with high costs.^4^ Detecting heart failure at an earlier stage could enable timelier interventions, help address disparities, and reduce disease progression, ultimately leading to decreased morbidity.^9^ However, despite the potential for symptoms to persist for several months, many initial heart failure diagnoses occur in acute care settings.^9^ Furthermore, non‐invasive monitoring of symptoms could facilitate the early detection of heart failure and development of efficient patient management strategies.^10^

Heart rate variability (HRV) is an indicator of the autonomic nervous system's activity.^11^, ^12^ It serves as a measurable marker for cardiovascular disease^13^ and other chronic diseases, including diabetes, inflammation, obesity, and psychiatric disorders.^12^, ^14^ HRV refers to the variation in time intervals between successive heartbeats, termed RR intervals (RRi). These intervals represent the time elapsed between two consecutive R‐waves of the QRS complex on the electrocardiogram (ECG).^11^ Congestive heart failure is often associated with autonomic dysfunction, which can be quantified through HRV measurements.^15^ Therefore, HRV has the potential to be an effective non‐invasive technique for detecting heart failure.

Machine learning models offer the potential to make significant contributions to early diagnosis by creating models capable of quantifying the complex physiological interactions between HRV and health risks.^16^ Machine learning methods have been applied to various heart failure‐related tasks, such as detection of heart failure from patient datasets, prediction of hospital readmissions, mortality prediction, and the classification and clustering of heart failure cohorts into subgroups with distinctive features and responses to heart failure treatments.^17^ However, there is a scarcity of studies examining the effectiveness of machine learning applied to HRV for the detection of individuals with heart failure. Therefore, this study aims to address this gap by evaluating the application of machine learning algorithms in distinguishing between healthy adults and individuals with congestive heart failure using HRV data derived from ECG signals.

## Methods

In this study, datasets consisting of ECG recordings from adult subjects with normal sinus rhythm and congestive heart failure were used for the experimental evaluation.

### Dataset

All the datasets used in this study are standard datasets available on the PhysioNet portal, which can be freely accessed for research purposes.^18^ Specific details regarding the datasets, including the normal sinus rhythm RRi database,^18^ the congestive heart failure RRi database,^18^ and the Beth Israel Deaconess Medical Centre (BIDMC) congestive heart failure database,^19^ are provided on the PhysioNet portal alongside the datasets.

The normal sinus rhythm RRi database includes beat annotation files for 54 adults (men: 30 and women: 24, age range: 28.5–76 years).^18^ The original ECG recordings were digitized at a rate of 128 samples per second, and the beat annotations were obtained through automated analysis with manual review and correction.^18^

The congestive heart failure RRi database includes the beat annotation files for 29 long‐term ECG recordings of adults (men: 8, women: 2, and gender unknown: 21, age range: 34–79 years) with congestive heart failure [the New York Heart Association (NYHA classes 1, 2, and 3)].^18^ The original ECG recordings were digitized at 128 samples per second, and the beat annotations were obtained through automated analysis manual review and correction.^18^

Finally, the BIDMC congestive heart failure database includes long‐term ECG recordings from 15 subjects (men: 11 and women: 4, age range: 22–71 years) with severe congestive heart failure (NYHA classes 3 and 4).^19^ The original ECG signals were digitized at 250 samples per second, with 12 bit resolution over a range of ±10 mV. The beat annotations were prepared using an automated detector and have not been corrected manually.^19^

*Table* *1* provides the details of the participants' demographics. The normal sinus rhythm comprises 54 adults (men: 30 and women: 24, age: 61.36 ± 11.52 years). The congestive heart failure ECG recordings were combined from the congestive heart failure RRi database^18^ and the BIDMC congestive heart failure database.^19^ The congestive heart failure comprises 44 adults (men: 19, women: 6, and gender unknown: 19, age: 55.51 ± 11.3 years). Participants are identified as ‘nsr0XX’ in the normal sinus rhythm RRi database, ‘chf2XX’ in the congestive heart failure RRi database, and ‘chfXX’ in the BIDMC congestive heart failure database, where ‘XX’ denotes the participant's sequence number, ranging from the first participant to the last participant in each specific dataset.

**Table 1:** Summary of participants' demographic values

Variable	Healthy	CHF
Participants	54	44
Mean age ± SD (years)	61.36 ± 11.52	55.51 ± 11.3
Male	30	19
Female	24	6
Unknown	—	19

CHF, congestive heart failure; SD, standard deviation.

The beat‐annotated ECG compressed files were downloaded from the open‐access database.^18^, ^19^ The WFDB software package, which is part of the freely available Physio Toolkit software, was used to perform operations on the extracted ECG annotated files.^18^ Initially, the compatibility of the ECG annotated files was checked. Subsequently, essential information such as age, gender, NYHA classification, and recording details were extracted. Each annotation file was read, and the corresponding RRi files were generated and grouped into healthy and congestive heart failure data for analysis.

### Data pre‐processing

Two common reasons for noise in ECG recordings are lack of contact between the sensor and the participant's skin and rapid movement by the participant, which can cause the sensor to either produce additional spikes or miss some spikes.^20^ To mitigate these issues and ensure data quality, 10 min of initial and final recordings was discarded from each recording, considering set‐up time. Also, ectopic beats were eliminated from the recordings, that is, RRi shorter than 300 ms (i.e. 200 b.p.m.) or longer than 1300 ms (i.e. 46 b.p.m.).^21^

The moving average algorithm with a window size of 10 neighbouring data points was chosen. This window size of 10 strikes a balance between reducing amplitude and variance, effectively smoothening the captured signals. It helps mitigate the effects of missing data points, abnormal values, and misidentified R peaks.^20^

### Signal processing and feature extraction

This study employed time‐domain HRV analysis, which is considered sufficient when compared with frequency‐domain analysis.^12^ Moreover, as standard HRV analysis is typically performed on 5 min of RRi series,^22^ the filtered RRi data were segmented into 5 min of series for all individual recording. To conduct the HRV analysis, the study utilized the ‘hrv’ module, a Python package designed for HRV analysis.^23^

Time‐domain analysis involved a collection of statistical metrics, including the root mean square of the successive difference in adjacent RRi (RMSSD; Equation 1), the standard deviation of RRi (SDNN, the NN stands for natural or sinus intervals; Equation 2), the standard deviation of the successive differences between successive RRi (SDSD; Equation 3), the number or percentage of RRi longer than 50 ms (NN50 and pNN50; Equation 4), and the average value of RRi [mean RR interval (mRRi); Equation 5].^23^, ^24^, ^25^

RMSSD reflects the beat‐to‐beat variance in heart rate and is the primary time‐domain measure used to assess changes reflected in HRV and represents the short‐term variability between RRi:

(1) $$\text{RMSSD}=\sqrt{\frac{1}{N-1}\sum_{j=1}^{N-1}{\left({\mathrm{RRi}}_{j+1}-{\mathrm{RRi}}_{j}\right)}^{2}},$$

where *N* is the count of RRi values and *RRi*_j_ is the *j*th RRi value.

The SDNN provides information on short‐ and long‐term variability of the signal and could predict both morbidity and mortality:

(2) $$\text{SDNN}=\sqrt{\frac{1}{N-1}\sum_{j=1}^{N}{\left({\mathrm{RRi}}_{j}-\bar{\mathrm{RRi}}\right)}^{2}},$$

where *N* is the count of RRi values, *RRi*_j_ is the *j*th RRi value, and $\bar{\mathrm{RRi}}$ is the average value of the RRi series.

The SDSD is the standard deviation of the successive difference between adjacent RRi.

(3) $$\text{SDSD}=\sqrt{\frac{\sum_{i=1}^{i=n-1}{\left(D_{i}-D_{\text{mean}}\right)}^{2}}{n-1}},$$

where *i* is the interval index, *n* is the number of total intervals, $D_{i}$ is the successive difference between RRi, and

$$D_{\text{mean}}=\frac{1}{n-1}\sum_{i=1}^{i=n-1}D_{i}.$$

The pNN50 quantifies the percentage of successive intervals differing over 50 ms (nRRi_50_) to the total number of RRi (nRRi):

(4) $$\mathrm{pNN}50=\frac{{\text{nRRi}}_{50}}{\text{nRRi}}\times 100.$$

The mean value of the RRi after pre‐processing is computed as

(5) $$\text{Mean}=\frac{1}{N}\sum_{i=1}^{N}{\mathrm{RR}}_{i},$$

where *N* is the number of elements in the RRi and *RRi* is the *i*th element in RR time series.

### Machine learning approaches

This study used supervised classification machine learning algorithms to classify healthy and congestive heart failure participants. Additionally, utilizing robust off‐the‐shelf software, including machine learning models, allows manufacturers to focus on developing the application software necessary to run device‐specific functions in a medical device.^26^ While deep learning shows promise and has yielded promising results, it still faces several unresolved challenges in the clinical healthcare application, including issues related to data volume, data quality, disease's varying nature, domain complexity, and interpretability.^27^ Therefore, given the potential benefits of the findings in developing a home‐based medical device for congestive heart failure screening, this study used off‐the‐shelf machine learning models with high interpretability suitable for small and medium datasets.

The classification problem was addressed by training algorithms on the HRV data, with all programming implemented using the Python Scikit‐learn library.^28^ The classification algorithms utilized in this study include support vector machine (SVM), k‐nearest neighbour (KNN), naïve Bayes (NB), and decision tree (DT).^25^ SVM creates a decision hyperplane for classification to separate different classes.^25^ In Scikit, SVM using C‐support vector classification was implemented.^29^, ^30^ KNN method selects the most common class among k ‘neighbours’ of the object, the Gaussian NB applies Bayes' theorem, and the DT model around a sequence of the Boolean queries.^30^

To assess the effectiveness of machine learning models, a cross‐validation is performed. Cross‐validation verifies how well the model could predict unseen data to determine whether the model is underfitting, over fitting, or well generalized.^31^ A common cross‐validation technique is *k*‐fold validation, where the parameter *k* indicates the number of folds or sections that a given dataset is split into. During each fold, the machine learning model is trained using *k* − 1 folds and validated using the remaining one fold, resulting in *k* scores (accuracy).^31^

This study used five‐fold cross‐validation techniques to evaluate the classification models. The folds were stratified based on computed 5 min HRV parameter; that is, HRV parameters computed for an individual's 5 min duration are completely in the training set or completely in the test set.^32^ The five‐fold cross‐validation experiments involved random splits into five folds. However, to address the challenge associated with severely imbalanced datasets that could cause some folds not containing elements from all classes, the stratified cross‐validation method is used. This method preserves the percentage of samples from majority and minority classes by splitting the dataset on *k* folds.^33^ The stratified five‐fold cross‐validation ensures that the proportion of instances (healthy and heart failure recordings) is preserved in each partition. Consequently, a model is expected to accurately predict previously unseen HRV parameters as healthy or congestive heart failure during the testing phase.

The hyperparameters for each classifier were optimized through random search with repeated five‐fold cross‐validation. Furthermore, to assess the prediction accuracy, the cross‐validation process was repeated five times for each model on the dataset. This repetition aimed to obtain reliable performance results and report the mean for each metric.^32^

### Evaluation

The prediction accuracy of classification models was determined using various performance criteria, including precision (equivalent to positive predictive value; Equation 6), recall (equivalent to sensitivity; Equation 7), specificity (Equation 8), F1 score (the harmonic mean of precision and recall; Equation 9), and overall accuracy (Equation 10).^34^, ^35^ To compute these performance parameters, weighted average measures were considered. Precision measures the relationship between the true positive (TP) predicted values and the false positive (FP) predicted values^35^ and is identical to the positive prediction value, indicating the classifier's confidence when it identifies a person with a disease.^36^

(6) $$\mathrm{Pr}e\text{cision}=\frac{\mathrm{TP}}{\mathrm{TP}+\mathrm{FP}}.$$

Recall is the ratio of the total number of correctly classified positive instances to the total number of positive instances; that is, it is the number of class x cases correctly classified as class x divided by the total number of class x cases.^35^ In two‐class settings, similar to this scenario, recall is equivalent to sensitivity, which means it is the number of correctly classified cases divided by the total number of cases.^36^

(7) $$\text{Recall}=\frac{\mathrm{TP}}{\mathrm{TP}+\mathrm{FN}},$$

where FN is a false negative.

Specificity is the correctly classified cases divided by the total number of cases.^36^

(8) $$\text{Specificity}=\frac{\mathrm{TN}}{\mathrm{TN}+\mathrm{FP}}.$$

The F1 score allows for the comparison of two models, whether they have low precision and high recall or vice versa, by utilizing the harmonic mean.^35^

(9) $$F1=\frac{2\;*\;\text{Precision}\;*\;\text{Recall}}{\text{Precision}+\text{Recall}}.$$

Accuracy is the rate of correctly classified instances.^35^

(10) $$\text{Accuracy}=\frac{\mathrm{TP}+\mathrm{TN}}{\mathrm{TP}+\mathrm{TN}+\mathrm{FP}+\mathrm{FN}},$$

where TN is a true negative.

The chosen performance metrics were selected to evaluate binary classifiers on imbalanced datasets because they provide more informative and less misleading results compared with specificity and receiver operating characteristic (ROC) plots.^37^ ROC plots are visual tools for assessing the performance of binary classification models, especially when evaluating their sensitivity and specificity across different decision thresholds. The area under the ROC curve (AUC) is a single metric that summarizes the performance of the model over all possible thresholds. An AUC value closer to 1 indicates better overall classification performance, while an AUC value close to 0.5 suggests a model that performs no better than random chance. Therefore, to provide additional insights, we have presented the ROC plots and AUC values for evaluation and comparison of classification performance among different models.

## Results

The dataset included both healthy adults and patients with congestive heart failure, with the latter classified according to the NYHA classification. The congestive heart failure group consisted of 4 NYHA class 1 adults (gender: unknown, age: 53 ± 14.44 years), 8 NYHA class 2 adults (gender: unknown, age: 52 ± 15.15 years), 17 NYHA class 3 adults (age: 57 ± 9 years), and 15 NYHA classes 3–4 adults (56 ± 11 years). Among the 17 NYHA class 3 adults, there were 8 males (age: 57 ± 10.58 years) and 2 females (age: 48.5 ± 14.84 years) and gender information was unknown for 7 individuals (age: 60 ± 5.2 years). Similarly, among the 15 NYHA classes 3–4 adults, 11 were males (age: 54.7 ± 13.39 years) and 4 were females (age: 59.25 ± 3.86 years).

For each participant, the recordings underwent pre‐processing, which involved removing the initial and final 10 min (referred to as set‐up time). Subsequently, any recording segments containing ectopic beats were eliminated. Following this pre‐processing, approximately 1173 h of recording data was retained for healthy subjects, while approximately 793 h of recording data was retained for participants with congestive heart failure. Among the congestive heart failure patients, around 525 h of recording data came from the congestive heart failure RRi database, and an additional approximately 268 h was sourced from the BIDMC congestive heart failure database. In total, these recording durations were divided into approximately 14 076 five‐minute segments for healthy participants and roughly 9516 five‐minute segments for participants with congestive heart failure.

*Table* *2* presents the computed statistical time‐domain HRV parameters. The feature set considered for our analysis includes statistical time‐domain HRV parameters, specifically RMSSD, SDNN, SDSD, pNN50, and mRRi.

**Table 2:** Summary of variables

Variable	Healthy	CHF	*t*‐test (*P* value)
ECG recording duration (h)			
Processed	1173.95	793.57	—
HRV measures			
RMSSD (ms)	35.18 ± 47.51	20.17 ± 27.72	0.0374
SDNN (ms)	95.42 ± 90.58	51.81 ± 53	0.0036
SDSD (ms)	43.59 ± 48.01	17.75 ± 26.4	0.0373
NN50 (count)	1.56 ± 1.72	1.69 ± 2.19	0.3866
pNN50 (%)	0.53 ± 0.82	0.63 ± 0.98	−0.532
mRRi (ms)	852.661 ± 144.49	727.06 ± 164.2	0.0001
MHR (b.p.m.)	76.22 ± 7.38	88.87 ± 12.81	<0.0000

CHF, congestive heart failure; ECG, electrocardiogram; HRV, heart rate variability; MHR, mean heart rate; mRRi, mean relaxation response (RR) interval; NN50, number of RR intervals longer than 50 ms; pNN50, percentage of RR interval longer than 50 ms; RMSSD, root mean square of the successive difference in adjacent RR interval; SDNN, standard deviation of RR interval; SDSD, standard deviation of the successive differences between successive RR intervals.

In the *t*‐test analysis, RMSSD, SDNN, SDSD, mRRi, and mean heart rate (MHR) were found to be significant at *P* < 0.05, whereas NN50 and pNN50 were not significant at *P* < 0.05. Specifically, there was a significant effect for RMSSD, *t*(96) = 1.8018, *P* = 0.037409, with healthy adults [mean (M) = 35.18, standard deviation (SD) = 47.51] scoring higher than congestive heart failure patients (M = 20.17, SD = 27.72). Additionally, a significant effect was observed for SDNN, *t*(96) = 2.74441, *P* = 0.003638, with healthy adults (M = 95.42, SD = 90.58) achieving higher scores than congestive heart failure patients (M = 51.81, SD = 53). Similarly, there was a significant effect for SDSD, *t*(96) = 1.80287, *P* = 0.037323, with healthy adults (M = 43.59, SD = 48.01) obtaining higher scores than congestive heart failure patients (M = 17.75, SD = 26.4).

There was no significant effect for NN50, *t*(96) = −0.28892, *P* = 0.386644, even though congestive heart failure patients (M = 1.69, SD = 2.19) obtained slightly higher scores than healthy adults (M = 1.56, SD = 1.72). Similarly, there was no significant effect for pNN50, *t*(96) = −0.532, *P* = 0.297999, despite congestive heart failure patients (M = 0.63, SD = 0.98) achieving slightly higher scores than healthy adults (M = 0.53, SD = 0.82). In contrast, there was a significant effect for mRRi, *t*(96) = 3.95611, *P* = 0.000074, with healthy adults (M = 852.661, SD = 144.49) obtaining significantly higher scores than congestive heart failure patients (M = 1.56, SD = 1.72). Additionally, a significant effect was observed for MHR, *t*(96) = −6.05886, *P <* 0.00001, even though congestive heart failure patients (M = 88.87, SD = 12.81) achieved higher scores than healthy adults (M = 76.22, SD = 7.38).

To the obtained HRV parameters, we applied four machine learning classification algorithms. *Table* *3* presents the classification accuracy of machine learning approaches when considering all congestive heart failure patients. The overall best performance was achieved by KNN and DT trained on HRV data with a time window length of 5 min, achieving an accuracy of 77%. The optimal hyperparameters DT used are best splitter, max_depth as 9, and criterion as gini. This model exhibited a precision of 0.78, recall of 0.77, specificity of 0.79, and an F1 score of 0.77. Similarly, KNN utilized optimal hyperparameters, such as weights as distance, setting the number of neighbours to 20, and using the Manhattan metric. This KNN model demonstrated a precision of 0.77, recall of 0.77, specificity of 0.77, and an F1 score of 0.76.

**Table 3:** Classification accuracy of machine learning approaches considering all congestive heart failure patients

	Precision	Recall	Specificity	F1 score	Accuracy
SVM	0.74	0.74	0.74	0.73	0.74
KNN	0.77	0.77	0.77	0.76	0.77
NB	0.69	0.47	0.43	0.38	0.47
DT	0.78	0.77	0.79	0.77	0.77

DT, decision tree; KNN, k‐nearest neighbour; NB, naïve Bayes; SVM, support vector machine.

SVM achieved an accuracy of 74% using optimal hyperparameters, such as rbf kernel, gamma set to ‘scale’, and C value of 200. This SVM model exhibited a precision of 0.74, recall of 0.74, specificity of 0.74, and an F1 score of 0.73. On the other hand, NB achieved an accuracy of 47% with var_smoothing set to 1e‐09. The NB model showed a precision of 0.69, recall of 0.47, specificity of 0.43, and an F1 score of 0.38.

**Figure 1:**
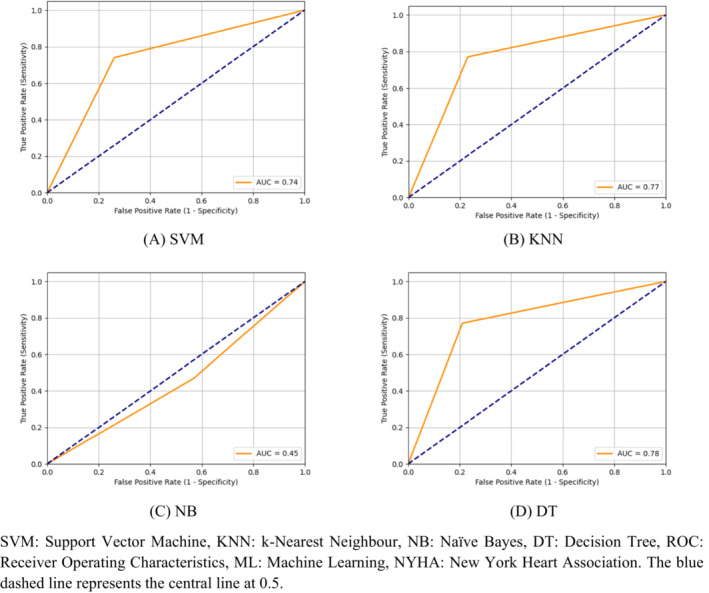
(A–D) Receiver operating characteristic (ROC) curves of machine learning approaches considering all congestive heart failure patients. The blue dashed line represents the central line at 0.5. AUC, area under the ROC curve; DT, decision tree; KNN, k‐nearest neighbour; NB, naïve Bayes; SVM, support vector machine.

*Figure* *1* presents the ROC curves for the various machine learning approaches when considering all congestive heart failure patients. SVM achieved an AUC of 0.74, indicating moderate discriminative ability in distinguishing between healthy and congestive heart failure patients. KNN performed slightly better with an AUC of 0.77, suggesting a relatively higher ability to classify the data correctly. In contrast, NB exhibited a poor AUC of only 0.45, suggesting that it struggled to effectively discriminate between the classes. DT, on the other hand, outperformed the other models, with the highest AUC of 0.78.

Heart failure recognition has the potential to reduce morbidity. Unfortunately, many cases of heart failure are diagnosed in acute care settings, after patients have already become clinically ill.^9^ The NYHA classification is a widely used method for assessing heart failure severity, but it has faced criticism due to its subjective nature.^38^ Therefore, in this study, the same machine learning classification algorithms are applied to classify participants as either healthy or suffering from congestive heart failure, based on NYHA classification. This approach is discussed in what follows.

In this experiment, we considered the congestive heart failure RRi database, which included patients from NYHA classes 1, 2, and 3 patients as the congestive heart failure patients. The congestive heart failure RRi database comprised 4 NYHA class 1, 8 NYHA class 2, and 17 NYHA class 3 patients. We also utilized the normal sinus rhythm RRi database for healthy adults. We applied the previously used machine learning classification algorithms to classify participants as either healthy or suffering from congestive heart failure. *Table* *4* summarizes the precision, recall, F1 score, and accuracy of the machine learning approaches for classifying healthy and congestive heart failure participants.

**Table 4:** Classification accuracy of machine learning approaches considering the congestive heart failure relaxation response interval database

	Precision	Recall	Specificity	F1 score	Accuracy
SVM	0.74	0.74	0.73	0.70	0.75
KNN	0.77	0.78	0.70	0.76	0.78
NB	0.69	0.37	0.32	0.27	0.37
DT	0.78	0.78	0.73	0.77	0.78

DT, decision tree; KNN, k‐nearest neighbour; NB, naïve Bayes; SVM, support vector machine.

KNN and DT achieved the highest accuracy of 78%. KNN exhibited a precision of 0.77, recall of 0.78, specificity of 0.70, and an F1 score of 0.76. Similarly, DT had a precision of 0.78, recall of 0.78, specificity of 0.73, and an F1 score of 0.77. SVM achieved an accuracy of 75% with a precision of 0.74, recall of 0.74, specificity of 0.73, and an F1 score of 0.70. On the other hand, NB had a lower accuracy of 37%, a precision of 0.69, recall of 0.37, specificity of 0.32, and an F1 score of 0.27.

**Figure 2:**
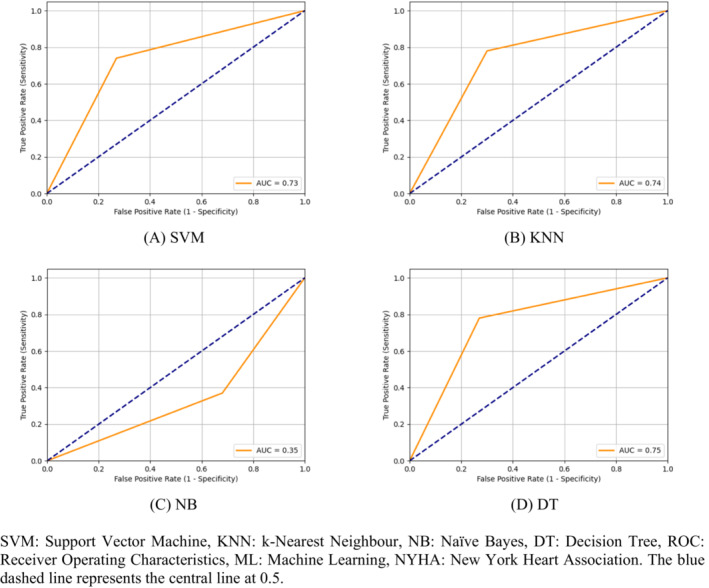
(A–D) Receiver operating characteristic (ROC) curves of machine learning approaches considering the congestive heart failure relaxation response interval database. The blue dashed line represents the central line at 0.5. AUC, area under the ROC curve; DT, decision tree; KNN, k‐nearest neighbour; NB, naïve Bayes; SVM, support vector machine.

*Figure* *2* displays the ROC curves for various machine learning approaches when considering patients from NYHA classes 1, 2, and 3. We observed that SVM achieved an AUC of 0.73, suggesting reasonable performance in distinguishing between the classes. KNN demonstrated a slightly improved AUC of 0.74, indicating a marginally better ability to classify data points. In contrast, NB exhibited a poor AUC of 0.35, suggesting severe limitations in its ability to effectively differentiate between the two classes. On a positive note, DT outperformed the other models with the highest AUC of 0.75.

In the subsequent experiment, we considered the BIDMC congestive heart failure database, which included 15 NYHA classes 3–4 patients as the congestive heart failure patients. Additionally, we utilized the normal sinus rhythm RRi database for healthy adults. We applied the same machine learning classification algorithms as before to classify participants as either healthy or suffering from congestive heart failure. *Table* *5* provides an overview of the precision, recall, F1 score, and accuracy of the machine learning classification algorithms in the classification of healthy and congestive heart failure participants.

**Table 5:** Classification accuracy of machine learning approaches considering the Beth Israel Deaconess Medical Centre congestive heart failure database

	Precision	Recall	Specificity	F1 score	Accuracy
SVM	0.90	0.90	0.82	0.89	0.90
KNN	0.91	0.91	0.85	0.90	0.91
NB	0.80	0.82	0.53	0.80	0.82
DT	0.90	0.91	0.92	0.90	0.91

DT, decision tree; KNN, k‐nearest neighbour; NB, naïve Bayes; SVM, support vector machine.

KNN and DT achieved the highest accuracy of 91%. KNN demonstrated a precision of 0.91, recall of 0.91, specificity of 0.85, and an F1 score of 0.90. Similarly, DT exhibited a precision of 0.90, recall of 0.91, specificity of 0.92, and an F1 score of 0.90. SVM achieved an accuracy of 90% with a precision of 0.90, recall of 0.90, specificity of 0.82, and an F1 score of 0.89. Finally, NB had an accuracy of 82% with a precision of 0.80, recall of 0.82, specificity of 0.53, and an F1 score of 0.80.

**Figure 3:**
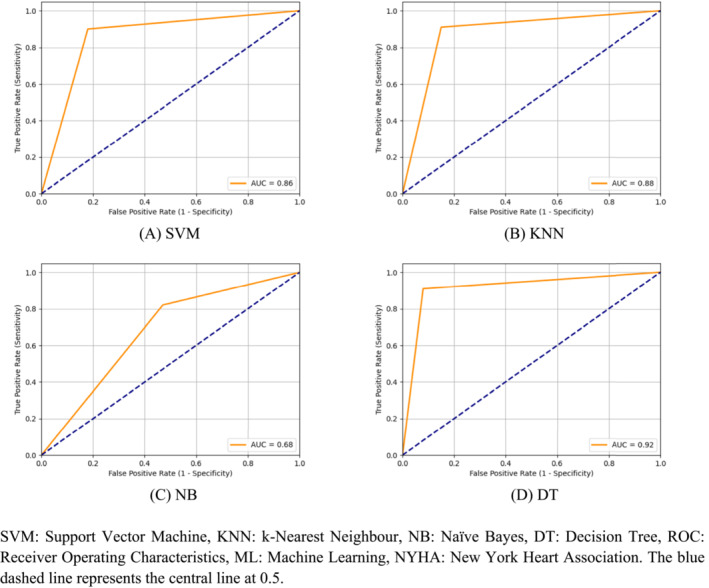
(A–D) Receiver operating characteristic (ROC) curves of machine learning approaches considering the Beth Israel Deaconess Medical Centre congestive heart failure database. The blue dashed line represents the central line at 0.5. AUC, area under the ROC curve; DT, decision tree; KNN, k‐nearest neighbour; NB, naïve Bayes; SVM, support vector machine.

*Figure* *3* depicts the ROC curves for different machine learning approaches when focusing on patients from NYHA classes 3 and 4. SVM achieved an AUC of 0.86, indicating its commendable ability to classify data accurately. Similarly, the KNN model demonstrated a strong AUC of 0.88, suggesting a robust capability to differentiate between classes. Conversely, NB exhibited a poor AUC of 0.68, signifying limitations in its effectiveness for classifying data points. In contrast, DT outperformed all other models, with the highest AUC of 0.92.

## Discussion

In this study, machine learning was applied to assess the potential of utilizing HRV computed from ECG signals for classification of healthy adults and individuals with congestive heart failure. The results indicate that off‐the‐shelf machine learning classification algorithms could classify healthy and congestive heart failure participants with an accuracy of 77% using the HRV parameters. The early detection of heart failure has the potential to enhance patient's quality of life through lifestyle modifications and necessary pharmacologic interventions that may slow disease progression.^39^ Furthermore, recognizing heart failure at an early stage could lead to reduction in both morbidity and mortality.^9^ However, heart failure typically has an insidious onset, progressing slowly for many years without evident symptoms, with symptoms becoming apparent only in the later stages of the disease.^40^ Therefore, there is a critical need for heart failure screening, identifying individuals at risk, and implementation of preventive measures to detect the condition at its initial stages.^40^ The findings from this study may contribute to development of non‐invasive tools for the early detection of heart failure among at‐risk populations.

Several studies have attempted to employ HRV in conjunction with machine learning for the purpose of heart failure detection. In a study involving 72 healthy (using MIT‐BIH normal sinus rhythm database) and 44 congestive heart failure patients (from the BIDMC database), an automated system to analyse HRV signals by extracting multimodal features to capture temporal, spectral, and complex dynamics was proposed.^41^ The study evaluated congestive heart failure detection performance based on single and hybrid features comprising time‐domain, frequency‐domain, and non‐linear measures and obtained the highest performance using DT with sensitivity (82%), specificity (82%), and accuracy (81.9%), and using SVM, the highest detection performance was obtained with SVM linear with sensitivity (96%), specificity (89%), and accuracy (93.1%).^41^ A review observed that studies showed greater consensus concerning time‐domain measures compared with frequency‐domain measures.^42^ Moreover, our study benefits from the simple methodology and explainability of the features utilized. Our study obtained the highest performance using DT with sensitivity (77%), specificity (79%), and accuracy (77%). Likewise, when using SVM, the highest detection performance was attained with sensitivity, specificity, and accuracy, all registering at 77%. Furthermore, considering NYHA classes 3–4 participants exclusively, our study obtained the highest performance using DT with sensitivity (91%), specificity (92%), and accuracy (91%). Similarly, using SVM led to the highest detection performance with sensitivity (91%), specificity (92%), and accuracy (91%). The findings suggest that HRV holds promise as a valuable non‐invasive predictor for the detection of severe congestive heart failure.

The application of machine learning on HRV has the potential to assist in categorizing patients based on the NYHA classification system. A prior study demonstrated that a combination of HRV indices and machine learning algorithms could accurately classify patients into NYHA functional classes 1, 2, and 3; however, the evaluation was conducted with a relatively small sample size of 29 participants.^43^ Affirmatively, this study reveals that the classification performance to classify NYHA classes 3–4 participants is higher when compared with classifying all congestive heart failure patients, including NYHA classes 1 and 2. These findings suggest that with further research and evaluation, HRV may hold the potential to effectively screen severe cases of heart failure.

Our study has limitations, and the results should be interpreted with caution. The healthy dataset consisted of 30 men and 24 women, while the congestive heart failure dataset had 19 men, 6 women, and missing gender details of 19 participants. The limited available data prevented us from conducting sex‐specific evaluations, which are essential for a more comprehensive understanding of the observed differences in prognosis among heart failure patients. This study could not explore these aspects due to participants' medical conditions being reported as unknown. Furthermore, our study utilized a combined dataset of 98 participants, with 55% being healthy and 45% having congestive heart failure. This sample size is relatively small, and it is important to recognize that larger datasets and randomized controlled trials would be beneficial for validating the machine learning algorithms for real‐world clinical utility, as suggested in previous research.^17^ Future studies with access to more extensive and diverse datasets can help overcome these limitations and provide more robust insights into the potential applications of machine learning in heart failure detection and classification. Current approaches to heart failure screening and treatment primarily rely on symptom‐based assessments, which often result in underdiagnosis and undertreatment of heart failure in various healthcare settings.^44^ Several factors contribute to the challenges in recognizing heart failure, including its misclassification as chronic obstructive pulmonary disease, deconditioning, age‐related symptoms, or obesity due to overlapping clinical presentations. Additionally, the unavailability of echocardiography in primary care further complicates timely diagnosis.^7^

These challenges underscore the need for more objective and rigorous management strategies that cover the entire spectrum of heart failure severity, ranging from mild to severe.^44^ Furthermore, there is growing interest in leveraging smart home technologies to monitor and manage heart failure patients within their home environments.^45^ Detecting heart failure at its earliest stages and harnessing the potential of the current technology to monitor patients at home present opportunities for the development of home‐based screening devices. The results of this study demonstrate the potential of machine learning in classifying healthy and those with congestive heart failure patients based on HRV analysis. This objective measure can help categorize patients according to the severity of their condition. The ease of capturing HRV data and the application of machine learning algorithms for classification and insights suggest that this research could have significant clinical utility. As HRV data can be readily collected, there is potential for this study to contribute to the improvement of congestive heart failure screening, monitoring, and management practices.

## Conclusions

In this study, using machine learning classification algorithms, we explored the potential of non‐invasive HRV to detect and manage heart failure patients. Our investigation involved 54 individuals with normal sinus rhythm and 44 congestive heart failure patients categorized under NYHA classes 1, 2, and 3. The study demonstrated that KNN and DT, when trained on HRV parameters with a 5 min time window, achieved the highest overall performance accuracy of 77%, with KNN achieving an AUC of 0.77 and DT attaining 0.78. For the classification of severe congestive heart failure, KNN and DT exhibited exceptional accuracy at 91%, with KNN achieving an AUC of 0.88 and DT obtaining an AUC of 0.92. These findings highlight the potential of HRV data and machine learning techniques as a non‐invasive biomarker for heart failure classification, offering it as a valuable tool for early detection and improved management in primary care settings. Also, as HRV data can be easily collected, there is potential for this study to contribute to the improvement of congestive heart failure screening, monitoring, and management practices.

## Conflict of interest

None declared.

## Funding

S.M.S.I. reports support for the present manuscript from an Emerging Leadership Fellowship from the National Health and Medical Research Council of Australia (APP1195406) and Vanguard grants from the National Heart Foundation of Australia; S.M.S.I. also reports unpaid leadership outside the submitted work with the IT Committee of the Cardiac Society of Australia and New Zealand, ESC Heart Failure Association Cardiac Devices Committee, and with WHO‐ITU Global Initiative on AI for Health. Open access publishing was facilitated by Deakin University, as part of the Wiley–Deakin University agreement via the Council of Australian University Librarians.

## References

[1] Zhang R , Ma S , Shanahan L , Munroe J , Horn S , Speedie S . Discovering and identifying New York Heart Association classification from electronic health records. BMC Med Inform Decis Mak 2018;18:48. 30066653 10.1186/s12911-018-0625-7 PMC6069768

[2] Bredy C , Ministeri M , Kempny A , Alonso‐Gonzalez R , Swan L , Uebing A , *et al*. New York Heart Association (NYHA) classification in adults with congenital heart disease: Relation to objective measures of exercise and outcome. Eur Heart J Qual Care Clin Outcomes 2018;4:51‐58. 28950356 10.1093/ehjqcco/qcx031

[3] Malik A , Brito D , Vaqar S , *et al*. Congestive Heart Failure (Nursing). Treasure Island (FL): StatPearls Publishing; 2021. 34662011

[4] Chen W , Liu G , Su S , *et al*. A CHF detection method based on deep learning with RR intervals. In: 2017 39th Annual International Conference of the IEEE Engineering in Medicine and Biology Society (EMBC); 2017. 10.1109/EMBC.2017.8037578 29060619

[5] Lippi G , Sanchis‐Gomar F . Global epidemiology and future trends of heart failure. AME Med J 2020;5:5.

[6] Watson RDS , Gibbs CR , Lip GYH . Clinical features and complications. BMJ 2000;320:236‐239. 10642237 10.1136/bmj.320.7229.236 PMC1117436

[7] Groenewegen A , Rutten FH , Mosterd A , Hoes AW . Epidemiology of heart failure. Eur J Heart Fail 2020;22:1342‐1356. 32483830 10.1002/ejhf.1858 PMC7540043

[8] Inamdar AA , Inamdar AC . Heart failure: Diagnosis, management and utilization. J Clin Med 2016;5:62. 27367736 10.3390/jcm5070062 PMC4961993

[9] Sandhu AT , Tisdale RL , Rodriguez F , Stafford RS , Maron DJ , Hernandez‐Boussard T , *et al*. Disparity in the setting of incident heart failure diagnosis. Circ Heart Fail 2021;14:e008538. 34311559 10.1161/CIRCHEARTFAILURE.121.008538 PMC9070116

[10] Faragli A , Abawi D , Quinn C , Cvetkovic M , Schlabs T , Tahirovic E , *et al*. The role of non‐invasive devices for the telemonitoring of heart failure patients. Heart Fail Rev 2021;26:1063‐1080. 32338334 10.1007/s10741-020-09963-7 PMC8310471

[11] Natarajan A , Pantelopoulos A , Emir‐Farinas H , Natarajan P . Heart rate variability with photoplethysmography in 8 million individuals: A cross‐sectional study. Lancet Digit Health 2020;2:e650‐e657. 33328029 10.1016/S2589-7500(20)30246-6

[12] Electrophysiology, T.F.o.t.E.S.o.C.t.N.A.S.o.P . Heart rate variability. Circulation 1996;93:1043‐1065. 8598068

[13] Singh N , Moneghetti KJ , Christle JW , Hadley D , Plews D , Froelicher V . Heart rate variability: An old metric with new meaning in the era of using mHealth technologies for health and exercise training guidance. Part one: physiology and methods. Arrhythmia Electrophysiol Rev 2018;7:193‐198. 10.15420/aer.2018.27.2 PMC6141929 30416733

[14] Young HA , Benton D . Heart‐rate variability: A biomarker to study the influence of nutrition on physiological and psychological health? Behav Pharmacol 2018;29:140‐151. 29543648 10.1097/FBP.0000000000000383 PMC5882295

[15] Nolan J , Batin PD , Andrews R , Lindsay SJ , Brooksby P , Mullen M , *et al*. Prospective study of heart rate variability and mortality in chronic heart failure. Circulation 1998;98:1510‐1516. 9769304 10.1161/01.cir.98.15.1510

[16] Chiera M , Cerritelli F , Casini A , Barsotti N , Boschiero D , Cavigioli F , *et al*. Heart rate variability in the perinatal period: A critical and conceptual review. Front Neurosci 2020;14:561186. 33071738 10.3389/fnins.2020.561186 PMC7544983

[17] Jasinska‐Piadlo A , Bond R , Biglarbeigi P , Brisk R , Campbell P , McEneaneny D . What can machines learn about heart failure? A systematic literature review. Int J Data Sci Analyt 2021;13:163‐183.

[18] Goldberger AL , Amaral LAN , Glass L , Hausdorff JM , Ivanov PC , Mark RG , *et al*. PhysioBank, PhysioToolkit, and PhysioNet. Circulation 2000;101:e215‐e220. 10851218 10.1161/01.cir.101.23.e215

[19] Baim DS , Colucci WS , Monrad ES , Smith HS , Wright RF , Lanoue A , *et al*. Survival of patients with severe congestive heart failure treated with oral milrinone. J Am Coll Cardiol 1986;7:661‐670. 3950244 10.1016/s0735-1097(86)80478-8

[20] Odenstedt Herges H , Vithal R , El‐Merhi A , *et al*. Machine learning analysis of heart rate variability to detect delayed cerebral ischemia in subarachnoid hemorrhage. Acta Neurol Scand 2022;145:151‐159. 34677832 10.1111/ane.13541

[21] Benchekroun M , Chevallier B , Istrate D , Zalc V , Lenne D . Preprocessing methods for ambulatory HRV analysis based on HRV distribution, variability and characteristics (DVC). Sensors (Basel) 2022;22:1984. 35271128 10.3390/s22051984 PMC8914897

[22] Baek HJ , Cho CH , Cho J , Woo JM . Reliability of ultra‐short‐term analysis as a surrogate of standard 5‐min analysis of heart rate variability. Telemed J E Health 2015;21:404‐414. 25807067 10.1089/tmj.2014.0104

[23] Bartels R , Peçanha T . HRV: A Pythonic package for heart rate variability analysis. J Open Source Softw 2020;5:1867.

[24] Bartels R , Neumamm L , Pecanha T , *et al*. SinusCor: An advanced tool for heart rate variability analysis. Biomed Eng Online 2017;16:110. 28923061 10.1186/s12938-017-0401-4 PMC5604194

[25] Kublanov VS , Dolganov AY , Belo D , *et al*. Comparison of machine learning methods for the arterial hypertension diagnostics. Appl Bionics Biomech 2017;2017:5985479. 28831239 10.1155/2017/5985479 PMC5555018

[26] de Hond AAH , Leeuwenberg AM , Hooft L , Kant IMJ , Nijman SWJ , van Os HJA , *et al*. Guidelines and quality criteria for artificial intelligence‐based prediction models in healthcare: A scoping review. NPJ Digit Med 2022;5:2. 35013569 10.1038/s41746-021-00549-7 PMC8748878

[27] Miotto R , Wang F , Wang S , Jiang X , Dudley JT . Deep learning for healthcare: Review, opportunities and challenges. Brief Bioinform 2018;19:1236‐1246. 28481991 10.1093/bib/bbx044 PMC6455466

[28] Brnabic A , Hess LM . Systematic literature review of machine learning methods used in the analysis of real‐world data for patient‐provider decision making. BMC Med Inform Decis Mak 2021;21:54. 33588830 10.1186/s12911-021-01403-2 PMC7885605

[29] Naicker N , Adeliyi T , Wing J , *et al*. Linear support vector machines for prediction of student performance in school‐based education. Math Probl Eng 2020;2020:1‐7.

[30] Pedregosa F , Varoquaux G , Gramfort A , *et al*. Scikit‐learn: Machine learning in Python. J Mach Learn Res 2011;12:2825‐2830.

[31] Elgeldawi E , Sayed A , Galal AR , Zaki AM . Hyperparameter tuning for machine learning algorithms used for Arabic sentiment analysis. Informatics 2021;8:79.

[32] Seo S , Kim Y , Han HJ , Son WC , Hong ZY , Sohn I , *et al*. Predicting successes and failures of clinical trials with outer product‐based convolutional neural network. Front Pharmacol 2021;12:670670. 34220508 10.3389/fphar.2021.670670 PMC8242994

[33] Szeghalmy S , Fazekas A . A comparative study of the use of stratified cross‐validation and distribution‐balanced stratified cross‐validation in imbalanced learning. Sensors (Basel) 2023;23:2333. 36850931 10.3390/s23042333 PMC9967638

[34] Yu H , Deng J , Nathan R , Kröschel M , Pekarsky S , Li G , *et al*. An evaluation of machine learning classifiers for next‐generation, continuous‐ethogram smart trackers. Mov Ecol 2021;9:15. 33785056 10.1186/s40462-021-00245-x PMC8011142

[35] Echtioui A , Zouch W , Ghorbel M , Mhiri C , Hamam H . Detection methods of COVID‐19. SLAS Technol 2020;25:566‐572. 32997560 10.1177/2472630320962002 PMC7533467

[36] Tohka J , van Gils M . Evaluation of machine learning algorithms for health and wellness applications: A tutorial. Comput Biol Med 2021;132:104324. 33774270 10.1016/j.compbiomed.2021.104324

[37] Matuz A , van der Linden D , Darnai G , Csathó Á . Generalisable machine learning models trained on heart rate variability data to predict mental fatigue. Sci Rep 2022;12:20023. 36414673 10.1038/s41598-022-24415-y PMC9681752

[38] Tripoliti EE , Papadopoulos TG , Karanasiou GS , Naka KK , Fotiadis DI . Heart failure: Diagnosis, severity estimation and prediction of adverse events through machine learning techniques. Comput Struct Biotechnol J 2017;15:26‐47. 27942354 10.1016/j.csbj.2016.11.001 PMC5133661

[39] Wang Y , Ng K , Byrd RJ , Hu J , Ebadollahi S , Daar Z , *et al*. Early detection of heart failure with varying prediction windows by structured and unstructured data in electronic health records. Annu Int Conf IEEE Eng Med Biol Soc 2015;2015:2530‐2533. 26736807 10.1109/EMBC.2015.7318907 PMC5233460

[40] Wilhelmsen L , Eriksson H , Svärdsudd K , *et al*. Improving the detection and diagnosis of congestive heart failure. Eur Heart J 1989;10:13‐18. 10.1093/eurheartj/10.suppl_c.13 2806286

[41] Hussain L , Awan IA , Aziz W , *et al*. Detecting congestive heart failure by extracting multimodal features and employing machine learning techniques. Biomed Res Int 2020;2020:4281243. 32149106 10.1155/2020/4281243 PMC7049402

[42] Shaffer F , Ginsberg JP . An overview of heart rate variability metrics and norms. Front Public Health 2017;5:258. 29034226 10.3389/fpubh.2017.00258 PMC5624990

[43] Qu Z , Liu Q , Liu C . Classification of congestive heart failure with different New York Heart Association functional classes based on heart rate variability indices and machine learning. Expert Syst 2019;36.

[44] Papadimitriou L , Moore CK , Butler J , Long RC . The limitations of symptom‐based heart failure management. Card Fail Rev 2019;5:74‐77. 31179015 10.15420/cfr.2019.3.2 PMC6546002

[45] Moses JC , Adibi S , Angelova M , Islam SMS . Smart home technology solutions for cardiovascular diseases: A systematic review. Appl Syst Innov 2022;5:51.

